# Autophagy Alters the Susceptibility of *Candida albicans* Biofilms to Antifungal Agents

**DOI:** 10.3390/microorganisms11082015

**Published:** 2023-08-05

**Authors:** Jiadi Shen, Ming Ma, Wei Duan, Yun Huang, Banruo Shi, Qiaochu Wu, Xin Wei

**Affiliations:** 1 Department of Endodontics, Affiliated Hospital of Stomatology, Nanjing Medical University, Nanjing 210000, China; shenjiadi1030@163.com (J.S.);; 2 Jiangsu Key Laboratory of Oral Diseases, Nanjing Medical University, Nanjing 210000, China

**Keywords:** *Candida albicans*, biofilm, antifungal agents, autophagy, drug resistance

## Abstract

*Candida albicans* (*C. albicans*) reigns as a major cause of clinical candidiasis. *C. albicans* biofilms are known to increase resistance to antifungal agents, making biofilm-related infections particularly challenging to treat. Drug resistance is of particular concern due to the spread of multidrug-resistant fungal pathogens, while autophagy is crucial for the maintenance of cellular homeostasis. Therefore, this study aimed to investigate the effects of an activator and an inhibitor of autophagy on the susceptibility of *C. albicans* biofilms to antifungal agents and the related mechanisms. The susceptibility of *C. albicans* biofilms to different antifungal agents after treatment with or without the autophagy activator or inhibitor was evaluated using XTT assay. Alkaline phosphatase (ALP) activity and reactive oxygen species (ROS) level, as well as the expression of ROS-related and autophagy-related genes, were examined to evaluate the autophagic activity of *C. albicans* biofilms when treated with antifungal agents. The autophagosomes were observed by transmission electron microscopy (TEM). The susceptibility of *C. albicans* biofilms to antifungal agents changed when autophagy changed. The ALP activity and ROS level of *C. albicans* biofilms increased with the treatment of antifungal agents, and autophagosomes could be observed in *C. albicans* biofilms. Autophagy was involved in the susceptibility of *C. albicans* biofilms to antifungal agents.

## 1. Introduction

*Candida albicans* (*C. albicans*) is a common opportunistic fungal pathogen in humans that can colonize the skin, mucosal surfaces, and gastrointestinal and vaginal tract of healthy people [1,2,3], while causing superficial mucosal infections or life-threatening systemic diseases in immunocompromised or immunologically deficient individuals [4]. Candidiasis leads to high incidence rates and mortality [5]. A major virulence attribute of *C. albicans* is that it exists in the form of biofilms in the human body [6]. Compared with the planktonic form, the biofilm form of *C. albicans* can escape an immune attack of the host and lead to persistent infection, which ultimately increases the difficulty of clinical treatment [7]. So far, *C. albicans* infection has been an important issue related to human health.

Currently, antifungal agents are mainly employed to treat *C. albicans* infection, and their sensitivity is related to their therapeutic effects [8]. Among them, four major classes of antifungal agents are commonly employed, including polyenes (like amphotericin B), azoles (like fluconazole), echinocandins (like caspofungin), and pyrimidine or nucleoside analogs (like flucytosine), which are fungicidal against *C. albicans* [9,10]. Nevertheless, with the increasing use of antifungal agents, drug resistance to conventional antifungal therapeutics makes *C. albicans* biofilm-associated infections and drug resistance significant clinical challenges [11,12].

Autophagy is a conserved cellular process in eukaryotic cells, which is associated with the virulence of *C. albicans* [13,14]. Moreover, autophagy regulation is involved in biofilm formation and antifungal resistance of *C. albicans* [15]. However, the effects of antifungal agents on the autophagic activity in *C. albicans* biofilms remain unknown. Autophagy is an essential process through which damaged organelles from cytoplasm are recycled to sustain the development of organisms [16,17], and it is more generally classified as a basic cell survival mechanism against environmental stressors [18]. The autophagic activity of eukaryotic cells can be analyzed through alkaline phosphatase (ALP) activity, reactive oxygen species (ROS) level, and the expressions of autophagy-related genes and proteins [15]. In the present study, susceptibility tests, ALP activity, ROS level, mitochondrial membrane potential (MMP), autophagosomes, and the expressions of autophagy-related genes and proteins were used to compare the autophagy level of *C. albicans* biofilms under treatment with different antifungal agents. This study has shed new light on how autophagy changes the susceptibility of *C. albicans* biofilms to antifungal agents and its related mechanisms.

## 2. Materials and Methods

### 2.1. Strain and Growth Media

The standard strain *Candida albicans SC5314* (*C. albicans SC5314*, ATCC^®^ MYA-2876TM) was purchased from the American Type Culture Collection (ATCC, Manassas, VA, USA). The strain was stored at −80 °C until use. A yeast extract peptone dextrose (YPD) medium (2% peptone, 1% yeast extract, and 2% glucose) was prepared for *C. albicans* strain incubation, and RPMI-1640 medium (Gibco Ltd., Paisley, UK) was used for *C. albicans* biofilm formation.

### 2.2. Biofilm Formation

*C. albicans* was cultured overnight at 30 °C and 200 rpm by shaking in a 10 mL YPD medium to reach a logarithmic growth phase [19]. Cells were harvested by centrifugation at 3000 rpm and washed with phosphate buffered saline (PBS). Then, the *C. albicans* cells were resuspended in fresh RPMI 1640 medium (Gibco Ltd., Paisley, UK) and the cell density was adjusted with a haemocytometer (1 × 10^6^ cells/mL) [20]. The suspensions were placed in 96-well plates or culture dishes (Thermo Fisher Scientific Inc., Waltham, MA, USA). The plates or culture dishes were then incubated at 37 °C for 24 h for *C. albicans* biofilm formation.

### 2.3. Preparation of Antifungal Agents

Stock solutions of fluconazole (10 mg/mL), itraconazole (10 mg/mL), terbinafine (10 mg/mL), amphotericin B (5 mg/mL), nystatin (10 mg/mL), and 5-fluorocytosine (10 mg/mL) (Sigma-Aldrich, St. Louis, MO, USA) were dissolved in dimethyl sulfoxide (DMSO). These antifungal agents were prepared in serial two-fold dilutions based on the protocol M27-A4 document from the Clinical and Laboratory Standards Institute (CLSI, Berwyn, PA, USA) (2017), and their final concentrations ranged from 1 to 8 μg/mL for amphotericin B and nystatin and from 64 to 1024 μg/mL for fluconazole, itraconazole, terbinafine, and 5-fluorocytosine. Then, *C. albicans* biofilms were submitted to incubation with these antifungal agents for 24 h and biofilm SMIC_50_ (Sessile Minimum Inhibitory Concentration 50%) was analyzed using XTT [2,3-bis-(2-methoxy-4-nitro-5-sulfophenyl)-2H-tetrazolium-5-carboxanilide] assay (Sigma-Aldrich, St. Louis, MO, USA) [21], where SMIC_50_ was defined as 50% inhibition of *C. albicans* biofilm metabolic activity compared with a drug-free control group [22].

### 2.4. Susceptibility Tests

XTT and antifungal agents were prepared for susceptibility tests with reference to Section 2.3. *C. albicans* biofilms were treated with autophagy activator (rapamycin; 100 and 200 nM) or autophagy inhibitor (chloroquine; 100 and 200 nM) (Sigma-Aldrich, St. Louis, MO, USA) for 2 h, washed with sterile PBS twice, and then incubated with antifungal agents for 24 h. The SMIC_50_ of *C. albicans* biofilm was analyzed using XTT [21], where absorbance was measured at 490 nm using a microplate reader (SpectraMax M2, Molecular Devices, Sunnyvale, CA, USA). The tests were repeated three times.

### 2.5. Alkaline Phosphatase Activity Assay

Alkaline phosphatase (ALP) activity, as an indicator for autophagy, can be detected by a microplate reader [23]. *C. albicans* biofilms inoculated on the surface of culture dishes were treated with antifungal agents at the concentration of SMIC_50_ for 6 h and 24 h, respectively. Then, the biofilms were harvested for ALP activity assay, which was assessed based on previous research [15]. ALP activity was quantitatively analyzed with an alkaline phosphatase assay kit (Beyotime Biotechnology, Shanghai, China) according to the manufacturer’s instructions. Absorbance was recorded at 405 nm using a microplate reader (SpectraMax M2, Molecular Devices, Sunnyvale, CA, USA). The assay was performed in triplicate.

### 2.6. Reactive Oxygen Species (ROS)

ROS level was evaluated with 2′,7′-dichlorofluorescein diacetate (DCFH-DA) [24]. *C. albicans* biofilms that were incubated in 96-well plates were treated with antifungal agents at the concentration of SMIC_50_ for 24 h. The control (with no treatment) and treated biofilms were stained with 10 μM DCFH-DA (Beyotime Biotechnology, Shanghai, China) at 37 °C for 1 h in the dark, and were subsequently washed with PBS three times to remove residual dye. The fluorescence value (excitation wave 488 nm and emission wave 520 nm) was investigated via a microplate reader. In addition, the biofilms were incubated for 24 h in 96-well plates before being treated with antifungal agents for 24 h at the concentration of SMIC_50_ for fluorescence observation. Fluorescence images were obtained by an inverted fluorescence microscope (Leica DMI3000B, Wetzlar, Germany). The experiment was repeated in triplicate sets.

### 2.7. Mitochondrial Membrane Potential (MMP)

The JC-1 staining was applied to assess the changes in MMP [25]. Double fluorescence staining of mitochondria (green fluorescent J-monomers and red fluorescent J-aggregates) was employed to monitor the changes in mitochondrial membrane potential. *C. albicans* biofilms were treated with antifungal agents at the concentration of SMIC_50_ for 24 h, and then the biofilms of each group were stained with JC-1 (Beyotime Biotechnology, Shanghai, China) at 37 °C for 30 min. The fluorescence images were observed by an inverted fluorescence microscope (Leica DMI3000B, Wetzlar, Germany). Besides this, red and green fluorescence values were measured by a microplate reader, and then the relative ratio of red/green fluorescence was calculated. The test was repeated in triplicate.

### 2.8. Transmission Electron Microscopy

The autophagosomes in biofilm cells were observed by transmission electron microscopy (TEM; Carl Zeiss, Oberkochen, Germany). *C. albicans* biofilms after treatment with antifungal agents for 24 h at the concentration of SMIC_50_ were harvested, washed twice with PBS, and fixed in stationary liquid (glutaraldehyde) overnight at 4 °C. Then, the biofilm cells were fixed with 1% osmium tetroxide for 2 h at 4 °C. Next, the cells were dehydrated successively through ethanol (30, 50, 70, 80, 95, and 100%) and embedded into epoxy resins followed by being sliced and stained. Eventually, the prepared sections were observed using TEM.

### 2.9. RT-qPCR

RNA-seq data were verified by real-time quantitative PCR (RT-*q*PCR) analysis. Control and treated biofilms under antifungal agents for 24 h at the concentration of SMIC_50_ were harvested and frozen in liquid nitrogen for RNA extraction. Extraction of total RNA was carried out using an RNA extraction Kit (Vazyme, Nanjing, China). The integrity of the RNA of each group was measured using nanodrop (NanoDrop One, Thermo Fisher Scientific Inc., Waltham, MA, USA), and then the extracted RNA was submitted to be reversely transcribed into cDNA using HiScript II RT SuperMix (Vazyme, Nanjing, China) according to the manufacturer’s instructions. The cDNA was used to analyze the expressions of ROS-related genes and autophagy-related genes, which was performed on a QuantStudio™ 7 Flex (Thermo Fisher Scientific Inc., Waltham, MA, USA) using ChamQ Universal SYBR qPCR Master Mix (Vazyme, Nanjing, China). The relative expressions of genes were determined using the 2^−ΔΔCt^ method [26]. *β-actin* served as an internal reference gene. The primers and sequences are listed in Table 1. Three biological replicates were prepared for each sample and the test was repeated three times.

### 2.10. Western Blotting

Total protein extracts from the control and treated biofilms under antifungal agents for 24 h at the concentration of SMIC_50_ were prepared using an immunoprecipitation protocol for western blotting [27]. Total protein concentration was quantified using a BCA protein assay kit (Beyotime Biotechnology, Shanghai, China). The protein samples were electrophoresed by 10% SDS-PAGE gels and transferred onto PVDF membranes (Bio-Rad Laboratories, Hercules, CA, USA). The membranes were incubated with primary antibodies (ATG7, ATG13, ATG17, and ATG27; 1:500 dilution) (Dia-An Biotech, Wuhan, China) overnight at 4 °C after blocking with 5% skimmed milk in TBST, followed by incubation with corresponding secondary antibodies for 1 h. Target protein bands were detected with a chemiluminescence imaging system (Merck & Co., Inc., Kenilworth, NJ, USA). GAPDH (Bioworld, Minneapolis, MN, USA) served as a reference protein. All operations were repeated in triplicate.

### 2.11. Statistical Analysis

After validating the equal variance assumption of the data, the statistical significance of differences in groups was compared by one-way ANOVA. Statistical analyses were performed using SPSS Statistics 22.0 Software (SPSS Inc., Chicago, IL, USA). *p* < 0.05 was regarded as statistically significant.

## 3. Results

### 3.1. Autophagy Activator and Inhibitor Affected the Drug Resistance of C. albicans Biofilms to Antifungal Agents

The drug resistance of *C. albicans* biofilms to antifungal agents changed after being autophagy activator and inhibitor treated (Table 2). Among them, fluconazole had the worst inhibitory effect on *C. albicans* biofilms (SMIC_50_ value > 512), while nystatin exhibited the best (SMIC_50_ value > 2) as compared to the groups without the treatment of rapamycin or chloroquine. After treatment with rapamycin (100 nM and 200 nM), the SMIC_50_ of *C. albicans* biofilms to fluconazole, itraconazole, terbinafine, 5-fluorocytosine, amphotericin B, and nystatin were significantly increased (*p* < 0.05). After treatment with chloroquine (200 nM), the SMIC_50_ of *C. albicans* biofilms to terbinafine, 5-fluorocytosine, amphotericin B, and nystatin significantly decreased (*p* < 0.05), while fluconazole and itraconazole showed no significant difference regardless of the concentration of chloroquine compared with those groups with no treatment (*p* > 0.05).

### 3.2. Antifungal Agents Changed the ALP Activity of C. albicans Biofilms

**Figure 1:**
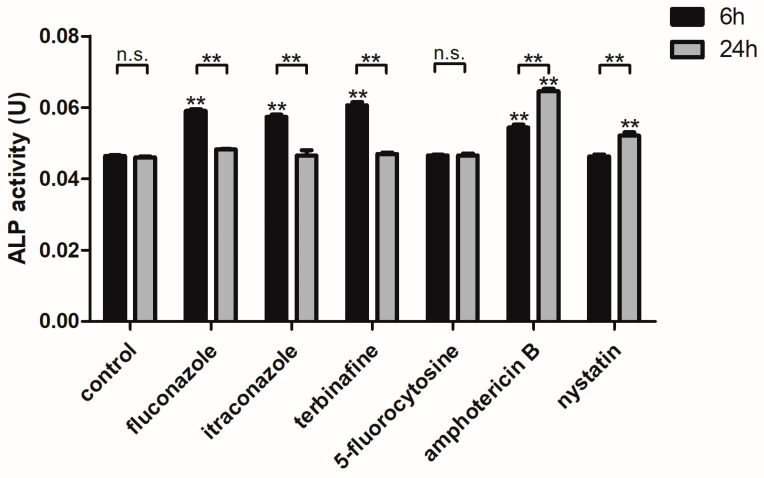
The ALP activity of *C. albicans* biofilms treated with antifungal agents for 6 h and 24 h. ** *p* < 0.01: ALP activity of *C. albicans* biofilms treated with antifungal agents for 6 h and 24 h compared with the control group; ** *p* < 0.01; n.s. no significant difference: comparison of ALP activity of *C. albicans* biofilms treated with antifungal agents between 6 h and 24 h.

The ALP activity of *C. albicans* biofilms significantly increased after the biofilms were treated with fluconazole, itraconazole, terbinafine, and amphotericin B (for 6 h) and with amphotericin B and nystatin (for 24 h) at the concentration of their SMIC_50_ (*p* < 0.01), respectively, while there was no significant difference in ALP activity of the groups treated with 5-fluorocytosine (for 6 h and 24 h) (*p* > 0.05) compared to the control group without antifungal agent treatment. Besides this, the ALP activity of *C. albicans* biofilms treated with fluconazole, itraconazole, and terbinafine for 6 h was higher than that for 24 h (*p* < 0.01), and the ALP activity of *C. albicans* biofilms treated with amphotericin B and nystatin for 24 h was higher than that for 6 h (*p* < 0.01) (Figure 1).

### 3.3. Antifungal Agents Increased the ROS Level of C. albicans Biofilms

**Figure 2:**
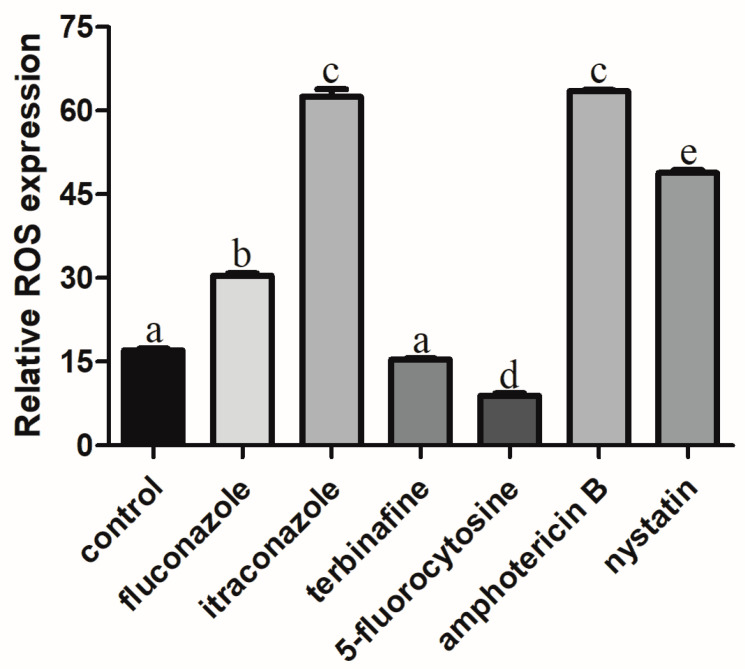
The ROS level of *C. albicans* biofilms treated with antifungal agents for 24 h. Values labeled with the same superscript are not significantly different (*p* > 0.05).

**Figure 3:**
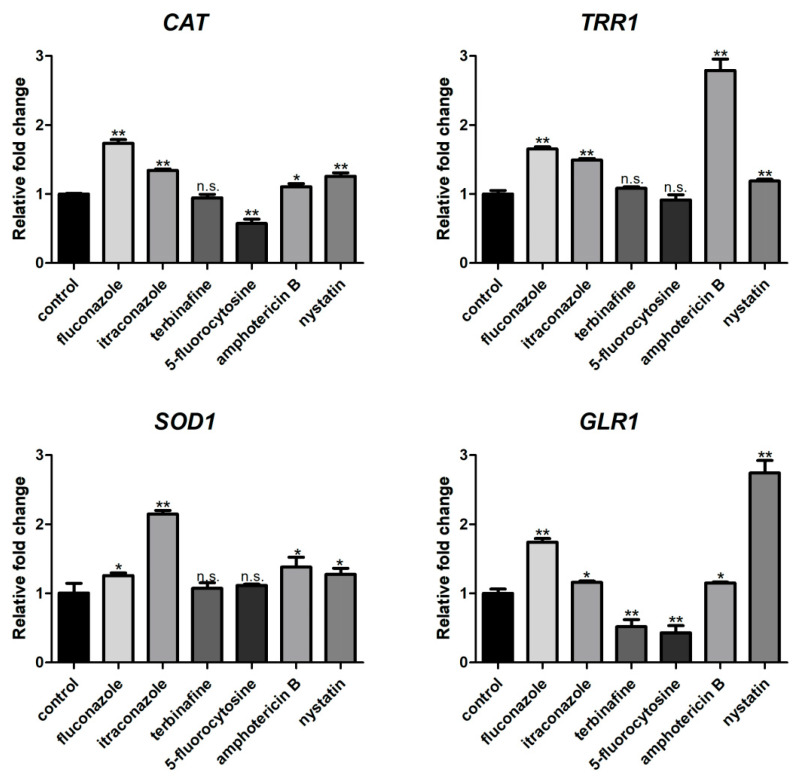
Expressions of ROS-related genes (*CAT*, *TRR1*, *SOD1*, and *GLR1*). * *p* < 0.05; ** *p* < 0.01; n.s. no significant difference.

**Figure 4:**
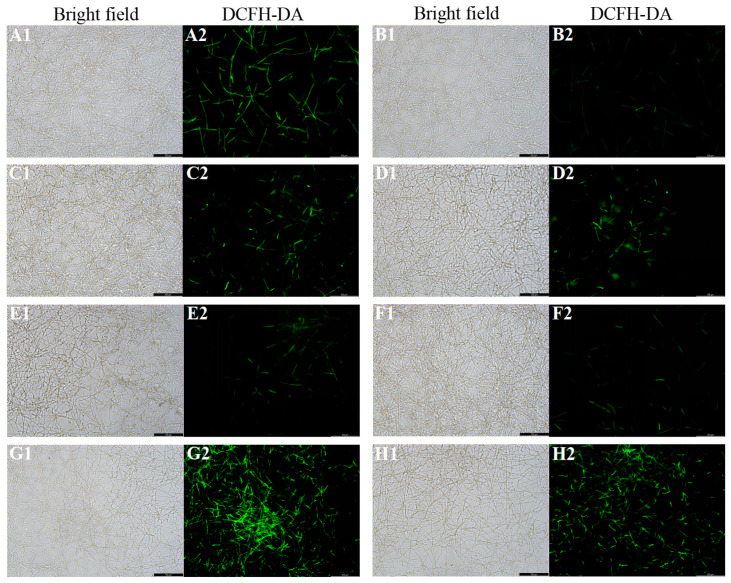
Representative DCFH-DA fluorescence images of *C. albicans* biofilms after treatment with antifungal agents for 24 h. (**A1**,**A2**): *C. albicans* ros up; (**B1**,**B2**): *C. albicans* (control); (**C1**,**C2**): fluconazole; (**D1**,**D2**): itraconazole; (**E1**,**E2**): terbinafine; (**F1**,**F2**): 5-fluorocytosine; (**G1**,**G2**): amphotericin B; (**H1**,**H2**): nystatin. Scale bar = 100 μm.

The ROS level of *C. albicans* biofilms significantly increased with the treatment of fluconazole, itraconazole, amphotericin B, and nystatin at the concentration of SMIC_50_ (*p* < 0.05) compared with the control group (Figure 2). In addition, the expressions of ROS-related genes (*CAT*, *TRR1*, *SOD1*, and *GLR1*) were significantly upregulated after treatment with fluconazole, itraconazole, amphotericin B, and nystatin (Figure 3), which was consistent with the results for ROS level (Figure 2). DCFH-DA fluorescence images showed that the green fluorescence intensity of *C. albicans* biofilms after treatment with fluconazole, itraconazole, amphotericin B, and nystatin was stronger than that of other groups (Figure 4).

### 3.4. Antifungal Agents Changed the MMP of C. albicans Biofilms

**Figure 5:**
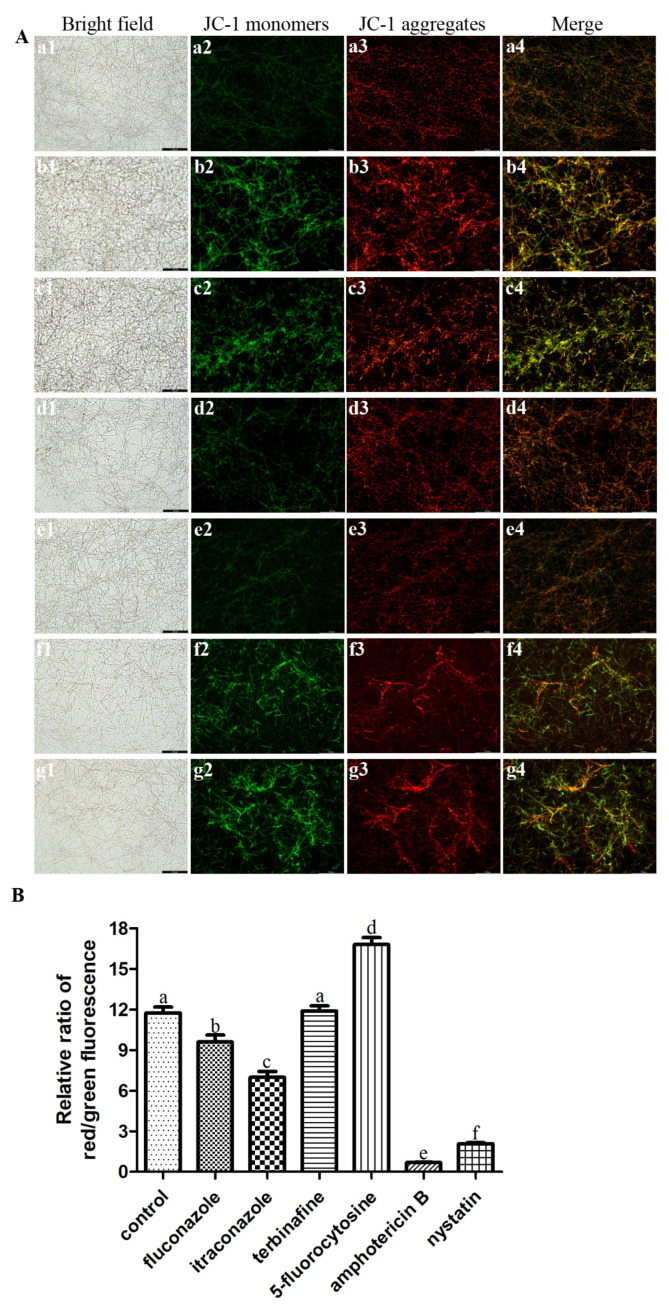
(**A**) Representative JC-1 fluorescence images of *C. albicans* biofilms after treatment with antifungal agents for 24 h. a: *C. albicans* (control); b: fluconazole; c: itraconazole; d: terbinafine; e: 5-fluorocytosine; f: amphotericin B; g: nystatin. Scale bar = 100 μm. (**B**) The relative ratio of JC-1 red/green fluorescence of *C. albicans* biofilms after treatment with antifungal agents for 24 h. Values labeled with the same superscript are not significantly different (*p* > 0.05). (**a1**–**g1**) Bright field. (**a2**–**g2**) JC-1 monomers. (**a3**–**g3**) JC-1 aggregates. (**a4**–**g4**) Merge.

To investigate whether ROS level affects mitochondrial function, the mitochondrial membrane potential (MMP) was measured to indicate the mitochondria functional status [28]. The JC-1 fluorescence images showed that the green fluorescence intensity of *C. albicans* biofilms after treatment with fluconazole, itraconazole, amphotericin B, and nystatin was stronger than that of other groups (Figure 5A), which was consistent with the ROS results above. Furthermore, the relative red/green fluorescence ratio of these groups as mentioned above (treatment with fluconazole, itraconazole, amphotericin B, and nystatin) decreased significantly (*p* < 0.05) (Figure 5B).

### 3.5. Antifungal Agents Increased the Autophagy Level of C. albicans Biofilms

**Figure 6:**
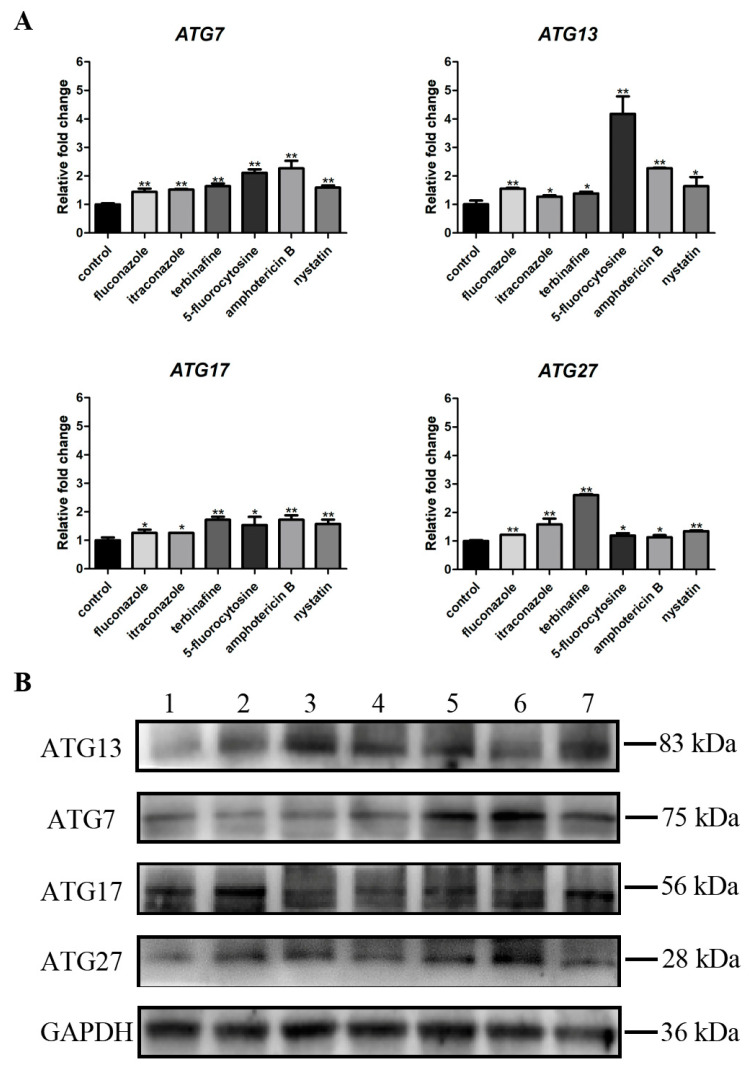
(**A**) Expressions of autophagy-related genes (*ATG7*, *ATG13*, *ATG17*, and *ATG27*). * *p* < 0.05; ** *p* < 0.01; (**B**) Protein expression levels of ATG7, ATG13, ATG17, and ATG27. 1: *C. albicans* (control); 2: fluconazole; 3: itraconazole; 4: terbinafine; 5: 5-fluorocytosine; 6: amphotericin B; 7: nystatin.

RT-qPCR and western blotting results presented changes in the autophagy level of *C. albicans* biofilms, where four critical autophagy-related genes (*ATG7*, *ATG13*, *ATG17*, and *ATG27*) were selected (Figure 6). RT-qPCR showed that the gene expressions of *ATG7*, *ATG13*, *ATG17*, and *ATG27* were significantly upregulated (*p* < 0.05) after treatment with different antifungal agents compared with the control group (Figure 6A). Moreover, western blotting results were consistent with RT-*q*PCR results that protein levels of ATG7, ATG13, ATG17, and ATG27 were higher in *C. albicans* biofilms treated with antifungal agents than the control group (Figure 6B).

### 3.6. Antifungal Agents Increased the Autophagosomes of C. albicans Biofilms

**Figure 7:**
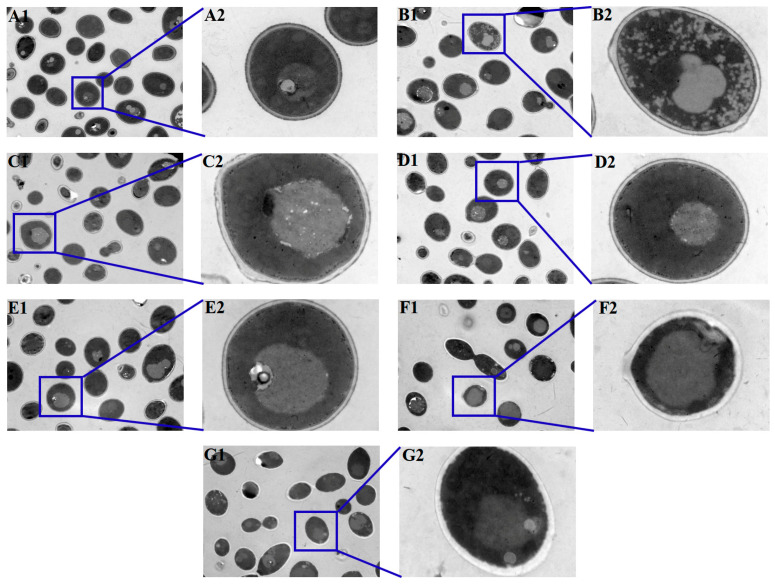
Observation of the autophagosomes in *C. albicans* biofilms after treatment with antifungal agents for 24 h by TEM. There were more autophagosomes in *C. albicans* antifungal-agent-treated biofilms, while fewer autophagosomes could be detected in the control group. (**A1**,**A2**): *C. albicans* (control); (**B1**,**B2**): fluconazole; (**C1**,**C2**): itraconazole; (**D1**,**D2**): terbinafine; (**E1**,**E2**): 5-fluorocytosine; (**F1**,**F2**): amphotericin B; (**G1**,**G2**): nystatin. ((**A1**–**G1**), 1.5 k× magnification; (**A2**–**G2**), 10 k × magnification).

Autophagosomes can be detected when autophagy occurs. Representative TEM images showed that *C. albicans* biofilms treated with antifungal agents exhibited more autophagosomes with distinct membranes and edges (Figure 7B1–G2), while fewer autophagosomes, despite having indistinct structures, could be observed in the control group (Figure 7A1,A2), indicating that treatment with antifungal agents increased the autophagosomes of *C. albicans* biofilms.

## 4. Discussion

Autophagy is a cellular degradation and recycling process through which damaged organelles and cellular components are recycled [18]. It plays an important role in maintaining intracellular homeostasis under nutrient depletion and stress conditions [29]. Nevertheless, the effects of autophagy on the susceptibility of *C. albicans* biofilms to antifungal drugs remain unclear, as well as the related autophagic molecular mechanism. The present study compared the susceptibility of *C. albicans* biofilms to different antifungal agents after treatment with or without an autophagy activator (rapamycin) or inhibitor (chloroquine), indicating that autophagy can affect the susceptibility of *C. albicans* to antifungal agents. The SMIC_50_ of *C. albicans* biofilms to all the antifungal agents used in this study increased after rapamycin treatment (*p* < 0.05); however, the SMIC_50_ of *C. albicans* biofilms to most antifungals (terbinafine, 5-fluorocytosine, amphotericin B, and nystatin) decreased after chloroquine treatment (*p* < 0.05). Autophagy activators (such as rapamycin), which can activate autophagy at low doses [30], may contribute to the resistance of *C. albicans* biofilms to antifungal agents. *C. albicans* engulfs damaged organelles through autophagy, thereby improving cell viability. Conversely, chloroquine inhibits autophagy by impairing autophagosome fusion with lysosomes [31,32,33], which may affect the efficacy of antifungal agents and decrease or not alter the drug resistance to some extent. Terbinafine has an antifungal effect by inhibiting squalene epoxidase, a key enzyme involved in ergosterol biosynthesis in the cell membrane of *C. albicans* [34]. The presence of terbinafine can result in the excessive accumulation of squalene and inhibit the synthesis of ergosterol [34]. Chloroquine destroys the lysosomal function of *C. albicans*, causing a faster accumulation of squalene. Consequently, the efficacy of terbinafine can be enhanced, which contributes to decreased SMIC_50_. Furthermore, 5-fluorocytosine can exert fungistatic effects by penetrating cells and interfering with the synthesis of nucleic acids [35]. After treatment with chloroquine, the permeability of lysosomes and mitochondria increases, and 5-fluorocytosine can more easily enter cells, which enhances its fungicidal effects, leading to a decrease in SMIC_50_. Several previous studies have reported that autophagy inhibitors (e.g., chloroquine) can result in a synergistic effect with chemotherapy in treating cancers [36]. The susceptibility results suggest that synergism between autophagy inhibitors and antifungal agents may be a potential way to treat *C. albicans* infections.

ALP activity was used to characterize autophagy levels [37]. In *Saccharomyces cerevisiae*, the precursor of ALP in cytoplasm is encapsulated in autophagosomes and then transported to vacuoles, in which it can be degraded by enzymes. Therefore, a subsequent increase in ALP activity will be detected when autophagy occurs [38]. The ALP activity of *C. albicans* biofilms increased after treatment with antifungal agents. Furthermore, the ALP activity of *C. albicans* biofilms treated with fluconazole, itraconazole, and terbinafine for 6 h was higher than that for 24 h, which may be due to the increased autophagy and adaptability of cells to these three agents after 24 h of treatment, ultimately resulting in the decrease in ALP activity. Amphotericin B and nystatin selectively bind to ergosterol in the cell membrane of *C. albicans* and increase its permeability [39,40]. With the prolongation of drug action time (for 24 h in this study), the permeability of cell membranes increases, which can be conducive to an increase in autophagy level. The findings suggested that the ALP activity of *C. albicans* biofilms may be affected by antifungal agents.

In addition, although the ALP activity value of *C. albicans* biofilms treated with 5-fluorocytosine was not statistically significant, the occurrence of autophagy could not be completely denied. The analysis of autophagy levels still needs to be determined with other detection methods such as ROS and MMP. Autophagy in yeast cells can be induced under stress stimulation or nutrient-deprived conditions, accompanied by changes in intracellular ROS levels [41,42]. Mitochondria is one of the main organelles of oxidative stress, in which mitochondrial respiratory complex I is one of the prominent sources of intracellular ROS [43,44]. ROS are highly reactive molecules containing oxygen, including peroxides, superoxides, and hydroxyl radicals (OH) [45,46]. High levels of ROS can activate autophagy in somatic cells, while autophagy can eliminate cell damage caused by ROS [47]. The ROS level of *C. albicans* biofilms treated with fluconazole, itraconazole, amphotericin B, and nystatin significantly increased compared with the control group, which was also clarified by previous studies that showed that antifungal treatment can induce ROS in fungal cells [48]. In *Aspergillus fumigatus*, antifungal agents affect the activity of mitochondrial respiratory complex I, resulting in excessive production of ROS, which indicates that ROS production is conducive to inhibiting the growth of fungi to some extent [49].

MMP depends on sequential intramitochondrial biochemical reactions [50]. Changes in MMP are one of the characteristics of mitochondrial dysfunction, which affects mitochondrial homeostasis [51]. In this study, a JC-1 fluorescent probe was used for evaluating MMP, in which aggregate (red) and monomer (green) forms allow dual-color, ratiometric assessment of MMP [52]. The relative red/green fluorescence ratio of *C. albicans* biofilms treated with fluconazole, itraconazole, amphotericin B, and nystatin decreased significantly (*p* < 0.05), indicating a decreased MMP for these groups. MMP regulates respiration rates and is associated with altered ROS levels [53,54], manifested as an increase in ROS production leading to a decrease in MMP [55,56]. Some antifungal agents like azoles can cause oxidative damage to the mitochondria of fungal cells such as *Cryptococcus neoformans* by increasing ROS levels [57,58].

Autophagosomes inside cells represent the occurrence of autophagy. TEM can observe autophagosomes in *C. albicans,* which is recognized as the “gold standard” in autophagy-related research [59]. In the present study, there were more autophagosomes in the *C. albicans* biofilms treated with different antifungal agents, while fewer autophagosomes were present in the control group, which indicated that the treatment with antifungal agents enhanced the autophagy response. Autophagosomes can be observed by high-precision TEM, which can identify intracellular structures as well as the internal environment and the surrounding cell components of autophagosomes [60].

Autophagy-related genes and proteins are also an important indicator of autophagic activity. Some autophagy-related genes (*ATG7*, *ATG13*, *ATG17*, and *ATG27*) have been identified in *C. albicans*, which were investigated in this research [15]. The expressions of these genes and proteins were upregulated in *C. albicans* biofilms when treated with antifungal agents compared with those in the control group, indicating that autophagic activity increased with treatment with antifungal agents.

In summary, autophagy changes affect the susceptibility of *C. albicans* biofilms to antifungal agents and also other parameters, such as ALP, ROS, and MMP. Autophagy levels (ALP) may be upregulated with an increase in ROS to reduce cellular stress and enhance the survival rate of *C. albicans*. Two *C. albicans* parameters (stress and survival) may exhibit upregulation or downregulation, depending on the treatment (activator versus inhibitor of the autophagy process). The regulation of autophagy levels in *C. albicans* biofilms may be a promising strategy to reduce the occurrence of clinical *C. albicans* drug resistance and improve the susceptibility to antifungal agents.

## 5. Conclusions

In the present study, the effects of autophagy on the drug resistance of *C. albicans* biofilms were preliminarily discussed. The autophagy level increased when *C. albicans* biofilms were treated with antifungal agents, and the autophagy was related to the drug susceptibility of *C. albicans*, suggesting that autophagy may be a regulatory mechanism of *C. albicans* drug resistance. Further studies are still needed to elucidate the prospective regulatory mechanisms underlying autophagy and drug resistance in *C. albicans*, as well as the molecular targets of antifungal agents.

## Figures and Tables

**Table 1:** Primers and sequences used in the present study.

Primer Name	Sequence (5′→3′)
*CAT*-F	CCAATTCCAGAACCATTTGCCACTC
*CAT*-R	ACCATAAGCACCGGAACCTTTAGC
*GLR1*-F	TGACAAGACTTTGATCGCCACTGG
*GLR1*-R	TCCAAGGCAAAGAACCCATCAGATG
*SOD1*-F	ACAAGAATCCGAATCCGCTCCAAC
*SOD1*-R	AGGACCAGCAGAAGTACAACCATTG
*TRR1*-F	GACCAACTCAAGACCGACGAAGC
*TRR1*-R	GCCATACATCCACTACCAGCAGAAG
*ATG7*-F	CTGGGGTGTCAGGAGCATTA
*ATG7*-R	GCATCTACACCGGGGAAAAC
*ATG13*-F	GCCAAGACTACGGGGTATGA
*ATG13*-R	AAGCATTGGAATTGCGTCGA
*ATG17*-F	TTCAACGCCTTCCAGCAA
*ATG17*-R	TGGTTTGATCTCTGGCATTGA
*ATG27*-F	ACTCCAACAGCTATCTCGCA
*ATG27*-R	TATAACGTCGCCAACCCT
*β-actin*-F	GACCAAGAAGACATCAAGGTATCAT
*β-actin*-R	GTGTTCAATTGGGTATCTCAAG

**Table 2:** The SMIC_50_ of *C. albicans* biofilms detected by XTT reduction assay.

Drugs	No Treatment (μg/mL)	Treated with 100 nM Rapamycin (μg/mL)	Treated with 200 nM Rapamycin (μg/mL)	Treated with 100 nM Chloroquine (μg/mL)	Treated with 200 nM Chloroquine (μg/mL)
fluconazole	>512	>1024 *	>1024 *	>512	>512
itraconazole	>256	>1024 *	>1024 *	>256	>256
terbinafine	>128	>512 *	>512 *	>128	>64 *
5-fluorocytosine	>256	>1024 *	>1024 *	>256	>128 *
amphotericin B	>4	>8 *	>8 *	>2 *	>2 *
nystatin	>2	>4 *	>4 *	>1 *	>1 *

* *p* < 0.05.

## Data Availability

The data presented in this study are available on request from the corresponding author.
